# Supplementary material for: Testing the Efficacy of RESPONSIBLEPLAY©: A Multi-Theory Model (MTM)-Based Intervention Protocol for Promoting Responsible Gambling Among College Students
Source: Int J Environ Res Public Health. 2025 May 30;22(6):858. doi: 10.3390/ijerph22060858 (PMC12193373; doi:10.3390/ijerph22060858)
Supplement: Supplementary file 1 [file ijerph-22-00858-s001.zip › ijerph-3611776-supplementary.pdf]

Supplement S1: RESPONSIBLEPLAY©: MTM-Based Intervention

Session One: Introduction to Responsible Gambling

Title: Promoting Responsible Gambling

Learning Objective	Content	Process	Time Frame
**Participatory Dialogue**			
Identify the benefits of responsible gambling.	Welcome participants and provide an overview of the session.<br><br>Introduce the concept of responsible gambling.<br><br>Discuss benefits such as financial stability, improved mental health, and better social relationships.	PowerPoint presentation.<br><br>Group discussion.<br><br>Flip charts.	15 minutes
Identify challenges associated with responsible gambling.	Discuss potential challenges such as efforts required to maintain responsible habits and deal with gambling urges.	Brainstorming session.<br><br>Materials: Flip charts	5 minutes
Highlight that the benefits outweigh the challenges.	Review the pros and cons.	Brainstorming session.	5 minutes
**Behavioral Confidence**			
Build confidence in maintaining responsible gambling.	Encourage participants to use their smartphones to take photos over the next week that depict responsible gambling behaviors, such as setting personal limits, taking breaks, and engaging in alternative leisure activities.<br><br>Participants will then share their photos and discuss the positive aspects they captured.	Photovoice activity.	15 minutes
Demonstrate responsible gambling strategies.	Explain what constitutes a responsible gambling session.	PowerPoint presentation.	
	Conduct a mini demonstration on setting personal limits and taking regular breaks.	Mini demonstration.	
**Changes in the Physical Environment**			

Identify changes in the environment to support responsible gambling.	Present strategies for avoiding gambling environments and resisting access to gambling resources.	PowerPoint presentation.	20 minutes
	Discuss practical changes like limiting access to funds for gambling and avoiding high-risk locations.	Discussion.	
Recap and address questions.	Summarize session content, address participants' questions, and outline next steps.	Group discussion.	
**Total**			60 minutes

Assignment After Session One:

- Complete Activity Sheet 1: Introduction to Responsible Gambling - Reflect on personal gambling habits and identify strategies for improvement.

Bring a list of personal strategies for maintaining responsible gambling behaviors to share in the next session.

Activity Guide for Session 1: Introduction to Responsible Gambling

Introduction

This activity guide is designed to assist both participants and facilitators in successfully completing Session 1. The guide provides detailed instructions and context for each activity, ensuring clarity and enhancing engagement.

…………………………………………………………………………………………………

Activity 1: Benefits of Responsible Gambling

Objective: Understand and identify the benefits of responsible gambling.

Instructions:

1. List three benefits of responsible gambling that you learned from the session.
2. Provide a brief explanation of why each benefit is important to you.

Tips for Facilitators:

- Use examples to illustrate each benefit and encourage participants to share their thoughts.
- Discuss how these benefits can positively impact their lives.

…………………………………………………………………………………………………

Activity 2: Challenges of Responsible Gambling

Objective: Recognize potential challenges in maintaining responsible gambling habits and brainstorm ways to overcome them.

Instructions:

1. List three challenges associated with maintaining responsible gambling habits that were discussed in the session.
2. Provide a brief explanation of how you might overcome each challenge.

Tips for Facilitators:

- Encourage open discussion about common challenges and brainstorm practical solutions as a group.
- Emphasize the importance of resilience and problem-solving.

……………………………………………………………………………………………………

Activity 3: Photovoice Activity

Objective: Use visual representation to express responsible gambling behaviors.

Instructions:

1. Use your smartphone to take a photo that represents responsible gambling behavior.
2. Describe what your photo depicts and how it relates to responsible gambling.

Tips for Facilitators:

- Explain the concept of photovoice and provide examples of what constitutes responsible gambling behaviors.
- Encourage creativity and personal expression.

……………………………………………………………………………………………………

Activity 4: Responsible Gambling Strategies

Objective: Identify and understand effective strategies for maintaining responsible gambling habits.

Instructions:

1. Write down two strategies for maintaining responsible gambling that you learned in the session.
2. Explain why these strategies are effective.

Tips for Facilitators:

- Discuss various strategies in detail and provide real-life examples.
- Highlight the importance of setting limits and taking regular breaks.

……………………………………………………………………………………………………

Activity 5: Environmental Changes

Objective: Recognize how changes in the physical can support responsible gambling.

Instructions:

1. List three changes you can make in your physical or social environment to support responsible gambling.
2. Explain how each change will help you maintain responsible gambling habits.

Tips for Facilitators:

- Provide examples of environmental changes, such as avoiding gambling venues or limiting access to funds.
- Encourage participants to think about their personal environments and make practical adjustments.

……………………………………………………………………………………………………

Facilitator Guide for Session 1: Introduction to Responsible Gambling

Introduction

This facilitator guide provides detailed instructions and talking points for conducting Session 1 of the RESPONSIBLEPLAY©: MTM-based intervention on responsible gambling. It is designed to help facilitators effectively deliver the session and engage participants in meaningful activities.

……………………………………………………………………………………………………

Start by welcoming participants and providing a brief overview of the session.

……………………………………………………………………………………………………

Activity 1: Benefits of Responsible Gambling

Explain the concept of responsible gambling and its importance. Discuss benefits such as:
- Financial stability: Explain how responsible gambling helps maintain financial health.
- Improved mental health: Discuss the positive effects on mental well-being.
- Better social relationships: Highlight how responsible gambling can improve relationships with family and friends.
Encourage participants to share their thoughts and experiences. Use a PowerPoint presentation and flip charts to illustrate key points.

……………………………………………………………………………………………………

Activity 2: Challenges of Responsible Gambling

Discuss common challenges such as:
- Efforts required to maintain responsible habits.
- Dealing with gambling urges.

Encourage open discussion and brainstorming on how to overcome these challenges. Use flip charts to capture participants' ideas. Emphasize resilience and problem-solving skills.

……………………………………………………………………………………………………

Activity 3: Photovoice Activity

Explain the concept of photovoice and its purpose in the session.
Provide examples of responsible gambling behaviors, such as setting limits or taking breaks.
Encourage creativity and personal expression. Allow time for participants to take photos and share them with the group. Facilitate a discussion on the photos and their significance.

……………………………………………………………………………………………………

Activity 4: Responsible Gambling Strategies

Discuss various strategies for responsible gambling, such as:
- Setting personal limits on time and money spent on gambling.
- Taking regular breaks to avoid continuous gambling.

Provide real-life examples and highlight the importance of these strategies. Encourage participants to share their thoughts and experiences.

……………………………………………………………………………………………………

Activity 5: Environmental Changes

Provide examples of environmental changes, such as:
- Avoiding gambling venues or situations that trigger gambling.
- Limiting access to funds that could be used for gambling.
- Seeking support from family and friends.

Encourage participants to think about their own environments and identify practical adjustments they can make. Facilitate a discussion on the impact of these changes.

……………………………………………………………………………………………………

Conclusion

Thank participants for their engagement in Session 1.
Encourage them to reflect on what they have learned and how they can apply it to their own lives.

……………………………………………………………………………………………………

Session 1 Worksheet: Introduction to Responsible Gambling

Activity 1: Benefits of Responsible Gambling

Instructions:
1. List three benefits of responsible gambling that you learned from the session.
2. Provide a brief explanation of why each benefit is important to you.

Benefits:

1. ____

2. ____

3. ____

……………………………………………………………………………………………………

Activity 2: Challenges of Responsible Gambling

Instructions:
1. List three challenges associated with maintaining responsible gambling habits that were discussed in the session.
2. Provide a brief explanation of how you might overcome each challenge.

Challenges:

1. ____

Overcoming Challenge:

____

2. ____

Overcoming Challenge:

____

3. ____

Overcoming Challenge:

____

……………………………………………………………………………………………………

Activity 3: Photovoice Activity

Instructions:
1. Use your smartphone to take a photo that represents responsible gambling behavior.
2. Describe what your photo depicts and how it relates to responsible gambling.

Photo Description:

____

____

……………………………………………………………………………………………………

Activity 4: Responsible Gambling Strategies

Instructions:
1. Write down two strategies for maintaining responsible gambling that you learned in the session.
2. Explain why these strategies are effective.

Strategies:

1. ____

Effectiveness: ____

2. ____

Effectiveness: ____

……………………………………………………………………………………………

Activity 5: Environmental Changes

Instructions:
1. List three changes you can make in your physical or social environment to support responsible gambling.
2. Explain how each change will help you maintain responsible gambling habits.

Environmental Changes:

1. ____

How it helps: ____

2. ____

How it helps: ____

3. ____

How it helps: ____

……………………………………………………………………………………………

Session Two: Understanding and Overcoming Barriers to Responsible Gambling

Title: Healthy Habits for Responsible Gambling

Learning Objective	Content	Process	Time Frame
**Emotional Transformation**			
Evaluate emotions associated with gambling.	Explore feelings related to gambling, both positive and negative.<br><br>Discuss how these emotions impact gambling behavior and the importance of emotional regulation.	Large group discussions.	15 minutes
**Practice for Change**			
Develop strategies to overcome barriers to responsible gambling.	Discuss common barriers such as peer pressure, financial stress, and boredom.<br><br>Participants brainstorm and share strategies to overcome these barriers.	Brainstorming session.<br><br>Chart paper.	10 minutes
Apply exercises to maintain responsible gambling behaviors.	Introduce journal writing as a tool to track gambling activities and emotions.	Journal writing.	20 minutes
	Conduct an interactive discussion on real-life scenarios and strategies to maintain responsible gambling.	Interactive discussion.	
**Changes in the Social Environment**			
Identify resources and support systems.	Discuss available resources such as counseling services, support groups, and responsible gambling apps.<br><br>Participants share personal experiences and resources they find helpful.	Group discussion.<br><br>Resource handouts.	15 minutes
**Total**			60 minutes

Assignment After Session Two:

- Identify a person who will support you in maintaining responsible gambling behaviors.

Complete Activity Sheet 2: Healthy Habits for Responsible Gambling - Reflect on barriers and strategies to overcome them.

Activity Guide for Session Two: Overcoming Barriers to Responsible Gambling

Introduction

This activity guide is designed to assist both participants and facilitators in successfully completing Session Two. The guide provides detailed instructions and context for each activity, ensuring clarity and enhancing engagement.

……………………………………………………………………………………………………

Activity 1: Review of Session One

Objective: Reinforce concepts covered in Session One and understand the benefits of responsible gambling.

Instructions:

1. Reflect on the key points discussed in Session One.
2. Write down the main takeaways about responsible gambling and its benefits.

Tips for Facilitators:

- Use examples to illustrate each benefit and encourage participants to share their thoughts.
- Discuss how these benefits can positively impact their lives.

……………………………………………………………………………………………………

Activity 2: Emotional Exploration

Objective: Recognize the emotional aspects of gambling and understand how feelings influence behavior.
Instructions:

1. List your positive and negative emotions associated with gambling.
2. Discuss how these emotions impact your gambling behavior.

Tips for Facilitators:

- Encourage open discussion about emotions related to gambling.
- Highlight the importance of emotional awareness in managing gambling behavior.

……………………………………………………………………………………………………

Activity 3: Brainstorming Strategies

Objective: Develop practical strategies to overcome barriers to responsible gambling.
Instructions:

1. Identify and list strategies to overcome barriers to responsible gambling.
2. Consider both personal and external barriers.

Tips for Facilitators:

- Encourage participants to think creatively and share their strategies.

- Discuss common barriers and brainstorm solutions as a group.

…………………………………………………………………………………………………

Activity 4: Journal Writing

Objective: Encourage self-reflection and provide a tool for tracking gambling behavior and emotions.
Instructions:

1. Start a journal entry to track your gambling activities and associated emotions.
2. Use the provided template.

Tips for Facilitators:

- Explain the benefits of journaling and provide examples.
- Encourage participants to be honest and detailed in their entries.

…………………………………………………………………………………………………

Activity 5: Overcoming Boredom

Objective: Provide strategies to manage boredom or frustration related to responsible gambling practices.
Instructions:

1. Discuss and list methods to deal with boredom or frustration.

Tips for Facilitators:

- Share practical examples and encourage participants to discuss their experiences.
- Highlight the importance of finding healthy alternatives to gambling.

…………………………………………………………………………………………………

Activity 6: Real-Life Scenarios

Objective: Prepare participants for real-world challenges by practicing problem-solving strategies.
Instructions:

1. Describe a real-life scenario where you might face a challenge to maintain responsible gambling.
2. Discuss how you would handle it.

Tips for Facilitators:

- Provide examples of common challenges and encourage group discussion.
- Emphasize practical solutions and resilience.

…………………………………………………………………………………………………

Activity 7: Resource Presentation

Objective: Inform participants about available resources for responsible gambling.
Instructions:

1. List resources available for responsible gambling (counseling services, support groups, apps).

Tips for Facilitators:

- Provide detailed information about each resource and encourage participants to seek support when needed.

……………………………………………………………………………………………

Activity 8: Support System Identification

Objective: Emphasize the importance of a support system in maintaining responsible gambling behaviors.
Instructions:

1. Identify a supportive person in your life who can help you maintain responsible gambling behaviors.
2. Discuss how this person can assist you.

Tips for Facilitators:

- Encourage participants to think about trusted individuals in their lives.
- Discuss the role of support systems in promoting responsible gambling.

……………………………………………………………………………………………

Activity 9: Emotional Transformation

Objective: Develop emotional coping strategies to support responsible gambling behaviors.
Instructions:

1. Write down techniques to transform negative emotions related to gambling into positive ones through responsible practices.

Tips for Facilitators:

- Provide examples of emotional transformation techniques.
- Encourage participants to share their own strategies and experiences.

……………………………………………………………………………………………

Activity 10: New Engagement Methods

Objective: Encourage creativity in finding new, healthier ways to engage in gambling activities.
Instructions:

1. Identify new ways to engage in responsible gambling.

Tips for Facilitators:

- Discuss various engagement methods and provide examples.
- Encourage participants to think about activities that align with responsible gambling principles.

……………………………………………………………………………………………………

Activity 11: Session Summary

Objective: Reinforce the session's content and ensure participants leave with a clear understanding of the material covered.
Instructions:

1. Summarize what you have learned in this session.
2. Address any questions or concerns.

Tips for Facilitators:

- Review the key points covered and encourage participants to ask questions.
- Ensure everyone has a clear understanding of the session's objectives.

……………………………………………………………………………………………………

Activity 12: Next Steps

Objective: Provide a clear roadmap for participants, helping them understand the progression of the intervention.
Instructions:

1. Outline the next steps and what participants can expect in the following sessions.

Tips for Facilitators:

- Explain the upcoming activities and objectives.
- Encourage participants to stay engaged and committed to the intervention.

……………………………………………………………………………………………………

Facilitator Guide for Session 2: Overcoming Barriers to Responsible Gambling

Introduction

This facilitator guide provides detailed instructions and talking points for conducting Session 2 of the RESPONSIBLEPLAY©: MTM-based intervention on responsible gambling. It is designed to help facilitators effectively deliver the session and engage participants in meaningful activities.
……………………………………………………………………………………………………
Start by welcoming participants and providing a brief overview of the session.

……………………………………………………………………………………………………

Activity 1: Review of Session One

Explain the importance of reinforcing concepts covered in Session One. Discuss the benefits of responsible gambling such as:
- Financial stability: Explain how responsible gambling helps maintain financial health.
- Improved mental health: Discuss the positive effects on mental well-being.
- Better social relationships: Highlight how responsible gambling can improve relationships with family and friends.

Encourage participants to share their thoughts and experiences. Use a PowerPoint presentation and flip charts to illustrate key points.

………………………………………………………………………………………………

Activity 2: Emotional Exploration

Discuss the emotional aspects of gambling and how feelings influence behavior. Explain the importance of recognizing both positive and negative emotions associated with gambling:
- Positive emotions: Euphoria, excitement, and thrill.
- Negative emotions: Anxiety, guilt, and frustration.

Encourage open discussion about these emotions. Highlight the importance of emotional awareness in managing gambling behavior. Use examples to illustrate key points.

………………………………………………………………………………………………

Activity 3: Brainstorming Strategies

Discuss common barriers to responsible gambling and have participants brainstorm strategies to overcome them. Examples of barriers include:
- Personal barriers: Lack of self-control, stress, and boredom.
- External barriers: Accessibility of gambling venues, peer pressure.

Encourage participants to share their strategies and discuss how they can implement these in their lives. Use flip charts to capture participants' ideas.

………………………………………………………………………………………………

Activity 4: Journal Writing

Introduce the concept of journal writing as a tool for tracking gambling activities and emotions. Explain the benefits of journaling:
- Self-reflection: Helps understand patterns and triggers.
- Emotional expression: Provides an outlet for feelings.

Provide a template for participants to start their journal entries. Encourage honesty and detail in their entries. Use examples to illustrate the process.

………………………………………………………………………………………………

Activity 5: Overcoming Boredom

Discuss strategies to manage boredom or frustration, which can be common triggers for gambling. Examples include:
- Engaging in hobbies: Reading, sports, arts.
- Social activities: Spending time with friends and family.

Encourage participants to share their own methods for overcoming boredom. Highlight the importance of finding healthy alternatives to gambling.

…………………………………………………………………………………………………

Activity 6: Real-Life Scenarios

Have participants describe real-life scenarios where they might face challenges in maintaining responsible gambling. Discuss problem-solving strategies:
- Identifying triggers: Recognize situations that lead to gambling.
- Developing action plans: Create steps to handle these situations.
Encourage group discussion and sharing of experiences. Emphasize practical solutions and resilience.

…………………………………………………………………………………………………

Activity 7: Resource Presentation

Present and discuss resources available for responsible gambling, such as counseling services, support groups, and apps. Examples include:
- National Gambling Helpline: Provide contact information.
- Local support groups: Share details of meetings and locations.
Encourage participants to seek support when needed. Provide detailed information about each resource.

…………………………………………………………………………………………………

Activity 8: Support System Identification

Discuss the importance of a support system in maintaining responsible gambling behaviors. Help participants identify supportive individuals in their lives:
- Family members: Spouse, parents, siblings.
- Friends: Close friends who understand and support their goals.
Discuss how these individuals can provide support. Encourage participants to reach out and seek assistance.

…………………………………………………………………………………………………

Activity 9: Emotional Transformation

Guide participants in writing down techniques to transform negative emotions related to gambling into positive ones. Examples of techniques include:
- Cognitive restructuring: Changing negative thought patterns.
- Relaxation techniques: Deep breathing, meditation.
Encourage participants to share their own strategies and experiences. Provide examples and facilitate a discussion on emotional coping.

…………………………………………………………………………………………………

Activity 10: New Engagement Methods

Encourage creativity in finding new, healthier ways to engage in gambling activities. Examples include:

- Setting time and money limits: Ensuring responsible play.
- Taking regular breaks: Avoiding continuous gambling.

Discuss various engagement methods and provide examples. Encourage participants to think about activities that align with responsible gambling principles.

…………………………………………………………………………………………………

Activity 11: Session Summary

Summarize the key points covered in the session. Ensure participants have a clear understanding of the material:
- Review the benefits of responsible gambling.
- Discuss strategies to overcome barriers.

Encourage participants to ask questions and share their thoughts. Thank them for their participation.

…………………………………………………………………………………………………

Activity 12: Next Steps

Provide a clear roadmap for participants, helping them understand the progression of the intervention. Outline the next steps:
- Preview upcoming activities and objectives.
- Encourage participants to stay engaged and committed.

Discuss how they can prepare for the upcoming sessions. Emphasize the importance of continued participation.

…………………………………………………………………………………………………

Session 2 Worksheet: Overcoming Barriers to Responsible Gambling

Activity 1: Review of Session One

Instructions:
1. Reflect on the key points discussed in Session One.
2. What are the main takeaways of responsible gambling and its benefits?

Key Takeaways:

1. ____
2. ____
3. ____

…………………………………………………………………………………………………

Activity 2: Emotional Exploration

Instructions:
1. List your positive and negative emotions associated with gambling.
2. How do these emotions impact your gambling behavior?

Positive Emotions:

1. ____
2. ____
3. ____

Negative Emotions:

1. ____
2. ____
3. ____

……

Activity 3: Brainstorming Strategies

Instructions:
1. Identify and list strategies to overcome barriers to responsible gambling.
2. Consider both personal and external barriers.

Strategies:

1. ____
2. ____
3. ____

……

Activity 4: Journal Writing

Instructions:
1. Start a journal entry to track your gambling activities and associated emotions.
2. Use the template below:

Date: ____

Gambling Activity: ____

Duration: ____

Amount Spent: ____

Emotions Felt (Positive/Negative): ______________________________

Reflections: ____

……

Activity 5: Overcoming Boredom

Instructions:
1. Discuss and list methods to deal with boredom or frustration that may arise from practicing responsible gambling.

Methods:

1. ____
2. ____
3. ____

……………………………………………………………………………………………………

Activity 6: Real-Life Scenarios

Instructions:
1. Describe a real-life scenario where you might face a challenge to maintain responsible gambling.
2. How would you handle it?

Scenario: ____

Strategy: ____

……………………………………………………………………………………………………

Activity 7: Resource Presentation

Instructions:
1. List resources available for responsible gambling (counseling services, support groups, apps).

Resources:

1. ____
2. ____
3. ____

……………………………………………………………………………………………………

Activity 8: Support System Identification

Instructions:
1. Identify a supportive person in your life who can help you maintain responsible gambling behaviors.
2. How can they assist you?

Supportive Person: ____

How They Can Help: ____

……………………………………………………………………………………………………

Activity 9: Emotional Transformation

Instructions:
1. Write down techniques to transform negative emotions related to gambling into positive emotions through responsible practices.

Techniques:

1. ____
2. ____
3. ____

……………………………………………………………………………………………………

Activity 10: New Engagement Methods

Instructions:
1. Identify new ways to engage in responsible gambling.

Methods:

1. ____
2. ____
3. ____

……………………………………………………………………………………………………

Activity 11: Session Summary

Instructions:
1. Summarize what you have learned in this session.
2. Do you have any questions or concerns?

……………………………………………………………………………………………………

Activity 12: Next Steps

Instructions:
1. Outline the next steps and what to expect in the following sessions.

Session Three: Sustaining Responsible Gambling Behaviors

Title: Responsible Gambling

**Learning Objective**	**Content**	**Process**	**Time Frame**
**Changes in the Social Environment**			
Identify a support person for responsible gambling.	Participants identify a family member, friend, healthcare professional, or community member who can support them in their responsible gambling efforts.<br><br>Discuss the importance of social support in sustaining responsible gambling behaviors.	Group discussion, guest speaker (e.g., from a local support group or health professional).	25 minutes
**Initiation of Responsible Gambling**			
Plan to initiate responsible gambling in the upcoming weeks.	Distribute various strategies and resources for initiating responsible gambling practices.<br><br>Discuss actionable steps participants can take in the next eight weeks.	Whole group discussion.<br><br>Resource handouts.	15 minutes
**Sustenance of Responsible Gambling**			
Develop strategies to sustain responsible gambling behaviors.	Discuss methods to maintain responsible gambling habits, including setting long-term goals and monitoring progress.<br><br>Participants share personal strategies and commit to specific actions.	Brainstorming session.<br><br>Commitment exercise.	10 minutes
Recap and preparation for follow-up.	Summarize session content, address participants' questions, and outline next steps, including the follow-up session in eight weeks.<br><br>Provide final encouragement and motivational tips.	Group discussion, Q&A	10 minutes
**Total**			60 minutes

Assignment After Session Three:

- Complete Activity Sheet 3: Develop a detailed plan for sustaining responsible gambling behaviors.
- Prepare for the eight-week follow-up session and be ready to share progress and challenges.

Activity Guide for Session 3: Sustaining Responsible Gambling Behaviors

Introduction

This activity guide is designed to assist both participants and facilitators in successfully completing Session 3. The guide provides detailed instructions and context for each activity ensuring clarity and enhancing engagement.

…………………………………………………………………………………………………

Activity 1: Identifying a Support Person

Objective: Identify a support person who can help you maintain responsible gambling behaviors.

Instructions:

1. Select a support person who can help you maintain responsible gambling behaviors.
2. Explain why you chose this person and how they can support you.

Tips for Facilitators:

- Use examples to illustrate how a support person can help.
- Encourage participants to think about someone they trust and who understands their goals.

…………………………………………………………………………………………………

Activity 2: Planning to Initiate Responsible Gambling

Objective: Develop a plan to initiate responsible gambling behaviors in the upcoming weeks.

Instructions:

1. List three strategies you will use to start responsible gambling.
2. Describe the resources you will need to implement these strategies.

Tips for Facilitators:

- Discuss various strategies in detail and provide examples.
- Highlight the importance of having a clear plan and the necessary resources.

…………………………………………………………………………………………………

Activity 3: Sustaining Responsible Gambling Behaviors

Objective: Develop strategies to sustain responsible gambling behaviors.

Instructions:

1. Write down two long-term goals for maintaining responsible gambling.
2. Explain how you will monitor your progress towards these goals.

Tips for Facilitators:

- Encourage participants to set realistic and achievable goals.
- Discuss methods of tracking progress, such as journals or apps.

…………………………………………………………………………………………………

Activity 4: Identifying Challenges and Solutions

Objective: Identify potential challenges in sustaining responsible gambling behaviors and brainstorm solutions.

Instructions:

1. Identify three potential challenges you might face in sustaining responsible gambling behaviors.
2. Provide possible solutions for each challenge.

Tips for Facilitators:

- Facilitate open discussion about common challenges.
- Encourage brainstorming and sharing of practical solutions.

…………………………………………………………………………………………………

Activity 5: Commitment to Responsible Gambling

Objective: Commit to specific actions to sustain responsible gambling behaviors.

Instructions:

1. Write a commitment statement about maintaining responsible gambling behaviors.

Tips for Facilitators:

- Encourage participants to think deeply about their commitment.
- Provide examples of strong commitment statements.

…………………………………………………………………………………………………

Activity 6: Follow-Up Preparation

Objective: Prepare for the eight-week follow-up session.

Instructions:

1. Reflect on the key takeaways from this session.

2. Plan what you will share about your progress and challenges in the eight-week follow-up session.

Tips for Facilitators:

- Discuss the importance of reflection and preparation for follow-up.
- Encourage participants to think about what they have learned and how they can continue to improve.

……………………………………………………………………………………………………

Facilitator Guide for Session 3: Sustaining Responsible Gambling Behaviors

Introduction

This facilitator guide provides detailed instructions and talking points for conducting Session 3 of the RESPONSIBLEPLAY©: MTM-based intervention on responsible gambling. It is designed to help facilitators effectively deliver the session and engage participants in meaningful activities.

……………………………………………………………………………………………………

Start by welcoming participants and providing a brief overview of the session.

……………………………………………………………………………………………………

Activity 1: Identifying a Support Person

- Discuss the importance of having a support person in maintaining responsible gambling behaviors.
- Provide examples of support persons such as a family member, friend, healthcare professional, or community member.
- Encourage participants to think about someone they trust and who understands their goals.
- Example: "Think about someone in your life who has always been supportive and understanding. How can they help you stay on track with your responsible gambling goals?"

……………………………………………………………………………………………………

Activity 2: Planning to Initiate Responsible Gambling

- Discuss various strategies for initiating responsible gambling such as setting a budget, limiting gambling time, and avoiding gambling when stressed.
- Highlight the importance of having a clear plan and the necessary resources to support these strategies.
- Provide examples of resources like budgeting apps, schedule planners, and relaxation techniques.
- Example: "Setting a budget can help you control your gambling expenses. What tools can you use to track your budget?"

……………………………………………………………………………………………………

Activity 3: Sustaining Responsible Gambling Behaviors

- Encourage participants to set realistic and achievable long-term goals.
- Discuss methods of tracking progress such as keeping a journal or using an app.
- Emphasize the importance of regularly reviewing and adjusting goals as needed.
- Example: "Long-term goals help keep you focused and motivated. What are some realistic goals you can set for yourself?"

……………………………………………………………………………………………………

Activity 4: Identifying Challenges and Solutions

- Facilitate open discussion about common challenges such as peer pressure, access to gambling websites, and emotional triggers.
- Encourage participants to brainstorm and share practical solutions.
- Provide examples of challenges and solutions.
- Example: "Peer pressure can be a big challenge. How can you set boundaries with your friends to avoid gambling?"

……………………………………………………………………………………………………

Activity 5: Commitment to Responsible Gambling

- Encourage participants to think deeply about their commitment and what it means to them.
- Provide examples of strong commitment statements.
- Discuss the importance of commitment in achieving long-term goals.
- Example: "A commitment statement helps reinforce your goals. What specific actions will you commit to?"

……………………………………………………………………………………………………

Activity 6: Follow-Up Preparation

- Discuss the importance of reflection and preparation for follow-up.
- Encourage participants to think about what they have learned and how they can continue to improve.
- Guide what to share during the follow-up session.
- Example: "Reflecting on your progress helps you stay motivated. What key takeaways will you share in the next session?"

……………………………………………………………………………………………………

Conclusion

Thank participants for their engagement in Session 3.
Encourage them to reflect on what they have learned and how they can apply it to their own lives.

……………………………………………………………………………………………………

ADDITIONAL MATERIALS RELATED TO RESPONSIBLE PLAY©: MTM-BASED INTERVENTION

Group Discussions

Allocated Duration: 15 minutes

Script and Directions

1. Introduction (3 minutes):
    - Facilitator: "Welcome everyone. Today, we will engage in a group discussion to explore the benefits and challenges of responsible gambling. Our goal is to understand different perspectives and build our confidence in practicing responsible gambling."
2. Participatory Dialogue (7 minutes):
    - Facilitator: "Let's start by discussing the advantages of responsible gambling. Please share your thoughts on how responsible gambling can positively impact your life. What are some benefits you can think of?"
    - Allow participants to share their views.
    - Facilitator: "Now, let's discuss some challenges or disadvantages you might face when trying to gamble responsibly. What makes it difficult to practice responsible gambling?"
    - Allow participants to share their views.
3. Behavioral Confidence Building (5 minutes):
    - Facilitator: "Let's talk about how we can overcome the challenges we just discussed. What strategies can we use to build our confidence in practicing responsible gambling?"
    - Encourage participants to share practical tips and personal experiences.

Role-Playing Scenarios

Allocated Duration: 20 minutes

Script and Directions

1. Introduction (3 minutes):
    - Facilitator: "Welcome everyone. Today, we will engage in role-playing scenarios to practice responsible gambling behaviors and manage our emotions effectively. Role-playing helps us prepare for real-life situations by acting them out in a safe environment."
2. Scenario 1: Managing Peer Pressure (10 minutes):
    - Facilitator: "In this scenario, you are at a social event where your friends are encouraging you to gamble more than you are comfortable with. How would you handle this situation?"
    - Divide participants into pairs. One person will play themselves, and the other will play the friend encouraging gambling.
    - Allow pairs to act out the scenario.
    - After 5 minutes, ask the pairs to switch roles and repeat the scenario.
3. Scenario 2: Controlling Urges to Gamble (7 minutes):

- Facilitator: "In this scenario, you are feeling a strong urge to gamble after a stressful day. How would you manage this urge and stay committed to responsible gambling?"
- Divide participants into pairs. One person will play themselves, and the other will play their inner voice urging them to gamble.
- Allow pairs to act out the scenario.
- After 3.5 minutes, ask pairs to switch roles and repeat the scenario.

Peer Feedback Sessions

Allocated Duration: 10 minutes

Script and Directions

1. Introduction (2 minutes):
   - Facilitator: "Welcome everyone. Today, we will engage in peer feedback sessions to provide constructive feedback and support each other in our journey towards responsible gambling. Constructive feedback helps us learn and grow by understanding our strengths and areas for improvement."
2. Feedback Round 1: Sharing Experiences (4 minutes):
   - Facilitator: "Let’s start by sharing our recent experiences with responsible gambling. What went well, and what challenges did you face?"
   - Divide participants into small groups of 3-4.
   - Allow each participant to share their experiences for 1 minute.
   - Encourage group members to listen actively and take notes.
3. Feedback Round 2: Providing Constructive Feedback (4 minutes):
   - Facilitator: "Now, let’s provide constructive feedback to each other. Focus on what your peer did well and suggest one or two areas for improvement."
   - Allow each group member to provide feedback to their peers.
   - Encourage participants to use positive and supportive language.

Activity Title: Photovoice Activity on Responsible Gambling Behaviors

Objective:

To encourage participants to identify and document responsible gambling behaviors through photography, and to share and discuss these behaviors with their peers to enhance understanding and behavioral confidence.

Materials Needed:

- Smartphone with a camera
- Notebook or digital device for jotting down notes
- Access to a computer or tablet for sharing photos (optional)

Instructions:

Introduction to Photovoice:

- What is Photovoice?
  Photovoice is a participatory research method where participants use photography to capture their experiences and perspectives on a particular topic. In this case, the focus is on responsible gambling behaviors.
- Purpose of the Activity:
  The goal is to help you reflect on and identify responsible gambling behaviors, understand their importance, and share your insights with the group.

Activity Duration:

This activity will take place over the next week. You will have 7 days to capture photos that represent responsible gambling behaviors.

Taking Photos:

Use your smartphone to take photos that depict responsible gambling behaviors. Examples include:

- Setting personal limits (e.g., setting a budget before gambling).
- Taking breaks during gambling sessions.
- Engaging in alternative leisure activities (e.g., sports, hobbies).
- Using tools or apps that promote responsible gambling.

Guidelines for Photos:

- Ensure the photos are clear and focus on the behavior you are capturing.
- You can take multiple photos to represent different aspects of responsible gambling.
- Be mindful of privacy. Avoid taking photos of people without their permission.

Journaling Your Experience:

Alongside taking photos, keep a journal of your thoughts and experiences. Note why you chose to take each photo, what it represents, and any reflections on the behavior depicted.

Preparation for Sharing:

- Select 2-3 photos that best represent responsible gambling behaviors.
- Prepare a brief description for each photo, explaining what it depicts and why you chose it.
- Think about the positive aspects of the behaviors you captured and how they contribute to responsible gambling.

Sharing Session (Google Drive):

- Share your photos and descriptions with the group.
- Discuss the positive aspects of the responsible gambling behaviors depicted in your photos.
- Reflect on what you have learned from this activity and how it has impacted your understanding of responsible gambling.

Submission:

If required, submit your selected photos and descriptions to the facilitator before the sharing with the group.

Reflection:

- After the sharing session, take some time to reflect on the insights gained from the activity and the group discussion.
- Consider how you can apply these responsible gambling behaviors in your own life.

Tips for Success:

- Be creative and thoughtful in capturing images that truly represent responsible gambling behaviors.
- Engage with the activity fully to gain the most from the reflective and sharing process.
- Respect others' privacy and obtain permission if you include people in your photos.

Contact Information:

- If you have any questions or need further assistance, please contact [Facilitator's Name] at [Facilitator's Contact Information].

Thank you for participating in the Photovoice Activity! Your contributions are valuable in promoting responsible gambling behaviors.

Activity Title: Mini Demonstration on Setting Personal Limits and Taking Regular Breaks

Objective:

To demonstrate practical strategies for setting personal limits and taking regular breaks during gambling sessions to promote responsible gambling behaviors.

Materials Needed:

- Whiteboard or flip chart and markers
- Timer or stopwatch
- Example materials (e.g., budget sheets, break schedules)
- Handouts on responsible gambling strategies (optional)

Instructions:

Introduction:

Purpose of the Demonstration:
This demonstration aims to provide practical examples and strategies for setting personal limits on gambling and taking regular breaks to promote responsible gambling behaviors.

Activity Duration:

The entire mini demonstration will take approximately 10 minutes.

Steps for Setting Personal Limits:

Explain the Importance:
Begin by explaining why setting personal limits is crucial for responsible gambling. Emphasize the benefits such as avoiding financial distress, maintaining control, and preventing excessive gambling.

- Financial Limits:

  Use a whiteboard or flip chart to show how to set a gambling budget. For example, demonstrate how to allocate a specific amount of money for gambling each week and how to stick to this budget.

  - Write down: "Weekly Gambling Budget: $50"
  - Explain that once this limit is reached, no more money should be spent on gambling for that week.
- Time Limits:
  Demonstrate how to set a time limit for gambling sessions. For example, use a timer or stopwatch to show setting a 1-hour limit for a gambling session.
  - Write down: "Gambling Session Time Limit: 1 hour"
  - Explain the importance of sticking to this limit to avoid prolonged gambling sessions.

Steps for Taking Regular Breaks:

Explain the Importance:
Discuss why taking regular breaks is essential. Highlight benefits such as reducing fatigue, maintaining focus, and making more mindful decisions.

- Demonstrate How to Schedule Breaks:
  - Use a timer or stopwatch to illustrate setting regular intervals for breaks. For example, set a timer for 30 minutes to remind gamblers to take a 5-minute break.
  - Write down: "Break Interval: Every 30 minutes"
  - Explain the importance of using these breaks to step away from the gambling environment, stretch, and engage in other activities.

Interactive Component:

Engage Participants:
Ask participants to share their strategies for setting limits and taking breaks. Encourage them to discuss what has worked for them in the past and any challenges they have faced.
Practice Setting Limits:
Distribute example budget sheets or break schedules and have participants practice setting their own limits. Provide guidance and feedback as needed.

Summary and Q&A:

- Summarize Key Points.

- Recap the importance of setting personal limits and taking regular breaks.
- Reinforce how these strategies can help maintain control and promote responsible gambling.

Questions and Answers:

Open the floor for any questions participants may have. Provide clear and concise answers to address any concerns or uncertainties.

Tips for Success:

- Be clear and concise in your explanations and demonstrations.
- Use visual aids effectively to enhance understanding.
- Encourage active participation and engagement from the participants.
- Provide practical examples that participants can relate to and implement in their own lives.

Activity Sheet 1: Introduction to Responsible Gambling

Title: Promoting Responsible Gambling

Instructions:

This activity sheet is designed to help you reflect on your current gambling habits and identify strategies for responsible gambling. Please answer the following questions honestly and thoughtfully. Your responses will help you develop a personalized plan for maintaining responsible gambling behaviors.

1. **What is the recommended amount of money you should gamble with?**

   Only what you can afford to lose

   More than you can afford to lose

   Any amount without limits

2. **How often should you take breaks while gambling?**

   Regularly

   Occasionally

   Never

3. **List four advantages of practicing responsible gambling.**

i. ____________________

ii. ____________________

iii. ______

iv. ______

4. List two disadvantages of not practicing responsible gambling.

i. ______

ii. ______

5. Identify two ways to increase your confidence in maintaining responsible gambling behaviors.

i. ______

ii. ______

6. What is one strategy to manage urges to gamble irresponsibly?

Avoid places where gambling occurs

Increase the amount of money gambled

Ignore urges and continue gambling

7. What is one step you can take to avoid environments that encourage irresponsible gambling?

Visit places where gambling is common

Limit access to funds for gambling

Spend more time in gambling venues

8. Who can support you in your goal of responsible gambling?

A family member or friend

A stranger you met at a casino

A person who encourages you to gamble more

9. Complete the following self-assessment questionnaire to evaluate your current gambling behavior. Answer each question honestly.

I. How often do you gamble?
   a. Daily
   b. Weekly
   c. Monthly
   d. Rarely

II. Do you often gamble more than you planned?
   a. Yes
   b. No

III. Have you ever borrowed money to gamble?
   a. Yes
   b. No

IV. Do you feel anxious or stressed about your gambling?
   a. Yes
   b. No

10. Review your responses and consider the following:

- Are there any patterns or concerns that stand out?
- How might these patterns affect your financial stability and well-being?

Reflection:

- How does your gambling behavior align with responsible gambling practices?
- What changes could you make to ensure you are gambling responsibly?

Activity Sheet 2: Healthy Habits for Responsible Gambling

Title: Understanding and Overcoming Barriers

Instructions:

This activity sheet is designed to help you identify barriers to responsible gambling and develop strategies to overcome them. Please answer the following questions honestly and thoughtfully. Your responses will help you create a personalized plan for maintaining healthy gambling habits.

1. **What are common barriers to practicing responsible gambling?**

   Peer pressure

   Financial stress

   Boredom

   All of the above

2. **Which of the following is an effective strategy to overcome peer pressure related to gambling?**

   Avoid gambling venues where friends are present

   Gamble more to fit in with friends

   Ignore your personal limits

3. **What is one way to manage financial stress to maintain responsible gambling?**

   Set a gambling budget and stick to it

   Use credit cards to gamble

   Borrow money to continue gambling

4. **How can journaling help in maintaining responsible gambling behaviors?**

   By tracking gambling activities and emotions

   By ignoring your gambling habits

   By encouraging you to gamble more frequently

5. **Which of the following resources can support responsible gambling?**

   Counseling services

   Support groups

   Responsible gambling apps

   All of the above

6. **List two strategies you can use to overcome feelings of boredom related to gambling.**

i. ______________________________

ii. ______________________________

7. **Identify one person who can support you in your goal of responsible gambling and describe how they can help.**
   - Person: ____
   - How they can help:
     ____

8. **Which of the following is a good practice to maintain responsible gambling behaviors?**

   Regularly review and adjust your gambling plan

   Ignore any changes in your gambling behavior

   Increase your gambling time gradually

Reflection:

- Reflect on the barriers you face in practicing responsible gambling. How can you address these barriers effectively?

What new strategies can you implement to ensure you maintain healthy gambling habits?

Activity Sheet 3: Sustaining Responsible Gambling Behaviors

Title: Responsible Gambling

Instructions:

This activity sheet is designed to help you develop a sustainable plan for responsible gambling. Please answer the following questions honestly and thoughtfully. Your responses will help you create a long-term strategy for maintaining responsible gambling habits.

1. **Who can support you in your goal of responsible gambling?**

   A family member

   A friend

   A healthcare professional

   All of the above

2. **What is one effective way to initiate responsible gambling practices in the upcoming weeks?**

   Set clear personal limits on gambling time and money

   Increase gambling frequency to test limits

   Avoid any planning and gamble spontaneously

3. **Which of the following strategies can help sustain responsible gambling behaviors?**

Regularly review and adjust your gambling plan

Ignore changes in gambling habits

Increase the amount of money gambled over time

4. **What should you do if you face challenges in maintaining responsible gambling behaviors?**

   Seek support from your identified support person

   Double your gambling efforts to overcome challenges

   Avoid discussing your struggles with anyone

5. **How can social support help in sustaining responsible gambling behaviors?**

   Provides encouragement and accountability

   Pressures you to gamble more

   Offers financial loans for gambling

6. **List two long-term goals for your responsible gambling behavior.**

i. ______________________________

ii. ______________________________

7. **Identify one strategy to overcome a potential barrier to responsible gambling in the next eight weeks.**
   - Strategy: ______________________________

8. **Which of the following is an important step for maintaining responsible gambling in the long term?**

   Continuously monitor and adjust your gambling habits

   Ignore any deviations from your plan

   Increase gambling stakes gradually over time

Reflection:

- Reflect on the strategies you have learned to sustain responsible gambling behaviors. How can you integrate these strategies into your daily routine?
- What additional support or resources do you need to maintain your responsible gambling habits over the long term?

Set aside 30 minutes each month to complete the following reflection exercise. Use the provided template to guide your reflections and adjustments.

Monthly Reflection Template

1. **Review Your Gambling Behavior:**
   - Frequency: How often did you gamble this month?
   - Amount Spent: How much money did you spend on gambling?
   - Time Spent: How many hours did you spend gambling?
2. **Evaluate Your Goals:**
   - Did you stick to your financial and time limits? (Yes/No)
   - What challenges did you face in meeting these limits?

3. **Assess Emotional Impact:**
   - How did gambling affect your mood and stress levels? (e.g., Did you feel anxious or stressed?)
   - Were there any signs of problematic behavior? (e.g., Feeling the need to gamble more, borrowing money)

4. **Adjust Your Plan:**
   - What changes will you make to your gambling goals or limits for the next month?
   - Do you need to seek additional support or resources?

Discussion:

- How can you address any challenges or signs of problematic behavior identified this month?
- What strategies can help you stay on track with your goals?

Supplement S2: Knowledge-Based Intervention

Session One: Introduction to Responsible Gambling

Title: Understanding Responsible Gambling

**Learning Objective**	**Content**	**Process**	**Time Frame**
Define responsible gambling.	Explain the concept of responsible gambling, emphasizing setting limits and taking breaks.<br><br>Discuss its importance in maintaining financial stability and mental health.	PowerPoint presentation.<br><br>Group discussion.	10 minutes
Discuss the significance of responsible gambling.	Highlight the risks of irresponsible gambling, including financial problems, health issues, and social consequences.	Lecture, Q&A.	15 minutes
Explain the benefits of responsible gambling.	Discuss benefits such as reduced stress, improved financial management, and better relationships.	Lecture.<br><br>Interactive discussion.	15 minutes
Identify common barriers to responsible gambling.	Discuss barriers like peer pressure, financial stress, and lack of awareness.	Brainstorming session.<br><br>Chart paper	10 minutes
Summarize key points and assign homework.	Recap session content and provide participants with an activity sheet to reflect on their gambling habits and identify strategies for improvement.	Group discussion.	10 minutes
**Total**			60 minutes

Assignment After Session One:

Complete Activity Sheet 1: Reflect on your gambling habits and identify strategies for responsible gambling.

Session Two: Overcoming Barriers to Responsible Gambling

Title: Strategies for Responsible Gambling

Learning Objective	Content	Process	Time Frame
Review key points from Session One.	Summarize the importance of responsible gambling, its benefits, and common barriers.	Group discussion.	10 minutes
Develop strategies to overcome barriers.	Discuss practical strategies to manage peer pressure, financial stress, and boredom.	Interactive lecture.<br><br>Group discussion.	20 minutes
Identify high-risk populations.	Discuss populations at higher risk for gambling problems, such as students, low-income individuals, and certain ethnic groups.	Lecture, Q&A.	10 minutes
Provide information on support systems and resources.	Discuss available resources such as counseling services, support groups, and responsible gambling apps.	Lecture. Resource handouts.	10 minutes
Create a personal action plan for responsible gambling.	Guide participants in developing a personalized plan to maintain responsible gambling behaviors.	Group activity. Individual planning.	10 minutes
**Total**			60 minutes

Assignment After Session Two:

- Complete Activity Sheet 2: Develop a detailed personal action plan for responsible gambling.

Session Three: Sustaining Responsible Gambling Behaviors

Title: Maintaining Responsible Gambling

**Learning Objective**	**Content**	**Process**	**Time Frame**
Review key points from Session Two.	Summarize strategies for overcoming barriers and the importance of support systems.	Group discussion.	10 minutes
Discuss long-term strategies for responsible gambling.	Highlight methods for maintaining responsible gambling habits, including setting long-term goals and regular self-assessment.	Interactive lecture.<br><br>Group discussion.	20 minutes
Emphasize the role of social support.	Discuss the importance of having a support system and how it can help sustain responsible gambling behaviors.	Lecture, Q&A.	15 minutes
Develop a final plan and prepare for follow-up.	Guide participants in finalizing their long-term plan and discuss the importance of follow-up sessions.	Group activity.<br><br>Individual planning.	15 minutes
**Total**			60 minutes

Assignment After Session Three:

- Complete Activity Sheet 3: Finalize your long-term responsible gambling plan and prepare for the follow-up session.

Activity Sheet 1: Introduction to Responsible Gambling

Title: Understanding Responsible Gambling

Instructions:

This activity sheet is designed to help you reflect on the concepts covered in Session One and understand the importance of responsible gambling. Please answer the following questions honestly and thoughtfully. Your responses will help reinforce the key points discussed in the session.

1. **What is the definition of responsible gambling?**

   Gambling within personal limits and taking regular breaks

   Gambling as much as possible

   Relying on gambling for income

2. **Why is responsible gambling important?**

   It helps maintain financial stability and mental health

   It increases the chances of winning big

   It allows you to spend more time at casinos

3. **List three benefits of practicing responsible gambling.**

i. ____

ii. ____

iii. ____

4. **Which of the following is a common barrier to responsible gambling?**

   Peer pressure

   Financial stress

   Lack of awareness

   All of the above

5. **What is one strategy to manage peer pressure related to gambling?**

   Avoid gambling venues where friends are present

   Gamble more to fit in with friends

   Ignore your personal limits

6. **What should you do if you experience urges to gamble irresponsibly?**

   Take a break and review your gambling plan

   Continue gambling until the urge passes

   Borrow money to keep gambling

7. **Identify one support resource you can use to help maintain responsible gambling behaviors.**

   Counseling services

   A stranger you met at a casino

   Online gambling forums

8. **Reflect on your current gambling habits. How do they align with the principles of responsible gambling?**

---

9. **What changes can you make immediately to start practicing responsible gambling?**

Reflection:

- Think about the information discussed in Session One. How can you apply these principles to your gambling habits?

What steps will you take to ensure you maintain responsible gambling behaviors?

Activity Sheet 2: Strategies for Responsible Gambling

Title: Overcoming Barriers to Responsible Gambling

Instructions:

This activity sheet is designed to help you reflect on the concepts covered in Session Two and develop strategies for responsible gambling. Please answer the following questions honestly and thoughtfully. Your responses will help reinforce the key points discussed in the session.

1. **What are common barriers to practicing responsible gambling?**

   Peer pressure

   Financial stress

   Boredom

   All of the above

2. **Which populations are at higher risk for gambling problems?**

   Students

   Low-income individuals

   Certain ethnic groups

   All of the above

3. **List two practical strategies to manage peer pressure related to gambling.**

i. ________________________________

ii. ________________________________

4. **What is one way to handle financial stress to maintain responsible gambling?**

   Set a gambling budget and stick to it

   Use credit cards to gamble

   Borrow money to continue gambling

5. **How can you incorporate responsible gambling practices into your daily routine?**

   Plan gambling activities in advance

   Gamble spontaneously without planning

Ignore personal limits

6. **Identify two resources or support systems that can help you maintain responsible gambling behaviors.**

i. ______

ii. ______

7. **What is one effective strategy to overcome boredom that leads to gambling?**

   Find alternative hobbies or activities

   Gamble more to pass the time

   Spend time at gambling venues

8. **Describe your personal action plan for maintaining responsible gambling behaviors.**

______

9. **How can regular self-assessment help in sustaining responsible gambling?**

   It helps monitor and adjust gambling habits

   It encourages more frequent gambling

   It has no impact on gambling habits

Reflection:

- Reflect on the barriers you face in practicing responsible gambling. How can you address these barriers effectively?

What new strategies can you implement to ensure you maintain healthy gambling habits?

Activity Sheet 3: Sustaining Responsible Gambling Behaviors

Title: Maintaining Responsible Gambling

Instructions:

This activity sheet is designed to help you reflect on the concepts covered in Session Three and develop a sustainable plan for responsible gambling. Please answer the following questions honestly and thoughtfully. Your responses will help reinforce the key points discussed in the session.

1. **What is a long-term strategy for maintaining responsible gambling behaviors?**

   Setting long-term goals and regularly assessing progress

   Ignoring changes in gambling habits

   Increasing the amount of money gambled over time

2. **Why is social support important in sustaining responsible gambling behaviors?**

   It provides encouragement and accountability

   It pressures you to gamble more

   It offers financial loans for gambling

3. **List two long-term goals you have for your responsible gambling behavior.**

i. ______________________________

ii. ______________________________

4. **What is one strategy to overcome a potential barrier to responsible gambling in the next eight weeks?**

   Seek support from your identified support person

   Double your gambling efforts to overcome challenges

   Avoid discussing your struggles with anyone

5. **How can you regularly assess and adjust your gambling habits?**

   Keep a self-record to monitor gambling behavior

   Ignore any deviations from your plan

   Increase gambling stakes gradually over time

6. **Identify one person who can support you in your goal of responsible gambling and describe how they can help.**
    - Person: ____
    - How they can help: ____

7. **What is an important step for maintaining responsible gambling in the long term?**

   Continuously monitor and adjust your gambling habits

   Ignore any changes in your gambling behavior

   Increase gambling time and money spent

8. **How can follow-up sessions help in sustaining responsible gambling behaviors?**

   They provide an opportunity to review progress and make necessary adjustments

   They encourage more frequent gambling

   They have no impact on gambling habits

9. **Describe your final plan for sustaining responsible gambling behaviors over the long term:** ____

Reflection:

- Reflect on the strategies you have learned to sustain responsible gambling behaviors. How can you integrate these strategies into your daily routine?
- What additional support or resources do you need to maintain your responsible gambling habits over the long term?

Supplement S3: Process Evaluation Tools

Reach Evaluation Tool

Responsible Gambling Behavior Promotion Program Evaluation

Directions:
Thank you for participating in our Responsible Gambling Behavior Promotion Program. Please help us improve our program by answering the questions below and circling the appropriate response. Your feedback is invaluable.

....

1. Participant Attendance (To be filled by the facilitator)
   Total number of participants: _______
   Session 1 attendance: _______
   Session 2 attendance: _______
   Session 3 attendance: _______

....

2. How old are you today? ___________________________ years

....

3. What sex were you assigned at birth (on your original birth certificate)?
   ☐ Male
   ☐ Female

....

4. What is your race or ethnicity?
   ☐ American Indian / Alaska Native, Alaskan
   ☐ Asian, Asian American
   ☐ Biracial, multiracial, of mixed race
   ☐ Black and African American
   ☐ Hispanic, Latino/a, Latinx, Latine
   ☐ Middle East, Middle Eastern or North African, Arab Americans
   ☐ White
   ☐Other,
   Please specify: ____________________________

....

5. What motivated you to join the program?
   ☐ Personal interest in gambling responsibly
   ☐ Recommendation from a healthcare provider
   ☐ Concern for a loved one
   ☐ Community involvement
   ☐Other,
   Please specify: ____________________________

....

6. How did you receive the invitation to participate in this program?
   ☐ Direct Email Invitation

☐ Weekly Student Newsletter Advertisement

7. Did you face any barriers to attending the sessions? (Check all that apply)
   ☐ Schedule conflicts
   ☐ Transportation issues
   ☐ Lack of interest
   ☐ None
   ☐Other,
   Please specify: ____________________________

8. How did these barriers affect your participation? (Select all that apply)
   ☐ Reduced frequency of attendance
   ☐ Prevented me from attending altogether
   ☐ Affected my level of engagement
   ☐ No significant impact

9. What aspects of the program did you find most helpful? (Select all that apply)
   ☐ Content and information provided
   ☐ Interaction with facilitators
   ☐ Supportive environment
   ☐ Flexibility of program delivery
   ☐Other,
   Please specify: ____________________________

10. What improvements would you suggest for the program? (Select all that apply)
   ☐ More flexible scheduling options
   ☐ Increased use of technology (e.g., better online tools)
   ☐ Enhanced outreach and marketing
   ☐ Additional support for overcoming barriers
   ☐ More engaging content or activities
   ☐Other,
   Please specify: ____________________________

11. Do you have any additional comments or suggestions for reaching more people and improving the program? ____

Thank you for your time!

Quality Evaluation Tool

Responsible Gambling Behavior Promotion Program Evaluation

Directions:

Thank you for participating in our Responsible Gambling Behavior Promotion Program. Please help us improve our program by answering the questions below and circling the appropriate response. Your feedback is invaluable.

1. How would you rate the adequacy of the time allocated for each session?

**Session 1**	**Session 2**	**Session 3**
☐ Poor	☐ Poor	☐ Poor
☐ Fair	☐ Fair	☐ Fair
☐ Good	☐ Good	☐ Good
☐ Excellent	☐ Excellent	☐ Excellent

2. How would you rate the relevance of the session content to your needs and interests?

**Session 1**	**Session 2**	**Session 3**
☐ Poor	☐ Poor	☐ Poor
☐ Fair	☐ Fair	☐ Fair
☐ Good	☐ Good	☐ Good
☐ Excellent	☐ Excellent	☐ Excellent

3. How would you rate the clarity and organization of the session content?

**Session 1**	**Session 2**	**Session 3**
☐ Poor	☐ Poor	☐ Poor
☐ Fair	☐ Fair	☐ Fair
☐ Good	☐ Good	☐ Good
☐ Excellent	☐ Excellent	☐ Excellent

4. How would you rate the facilitators' responsiveness to questions and concerns?

**Session 1**	**Session 2**	**Session 3**
☐ Poor	☐ Poor	☐ Poor
☐ Fair	☐ Fair	☐ Fair
☐ Good	☐ Good	☐ Good
☐ Excellent	☐ Excellent	☐ Excellent

5. How would you rate the usability of the materials provided during the sessions?

**Session 1**	**Session 2**	**Session 3**
☐ Poor	☐ Poor	☐ Poor
☐ Fair	☐ Fair	☐ Fair
☐ Good	☐ Good	☐ Good
☐ Excellent	☐ Excellent	☐ Excellent

…………………………………………………………………………………………………

6. How would you rate the atmosphere of the sessions in terms of being supportive and non-judgmental?

Session 1	Session 2	Session 3
☐ Poor	☐ Poor	☐ Poor
☐ Fair	☐ Fair	☐ Fair
☐ Good	☐ Good	☐ Good
☐ Excellent	☐ Excellent	☐ Excellent

…………………………………………………………………………………………………

7. Overall, how satisfied are you with the quality of the program? (*Only fill this after the whole program is ended*)

   ☐ Poor ☐ Fair ☐ Good ☐ Excellent

…………………………………………………………………………………………………

8. Do you have any additional comments or suggestions to enhance the quality of the program? ____

…………………………………………………………………………………………………

Thank you for your time!

Fidelity Evaluation Tools

RESPONSIBLEPLAY©: MTM-based Intervention: Promoting Responsible Gambling – Session One

Participatory Dialogue, Behavioral Confidence, and Change in Physical Environment

Objective: To evaluate the degree of implementation of the first session of the MTM-based intervention. The program evaluator will utilize this tally sheet to record observed actual behaviors as guided by the planned program.

Observer Name: ______________________

#	Item	Check If Performed	Time Planned	Time upon completion
1	Welcome participants and provide an overview of the session.	☐	2 min	
2	Define responsible gambling, emphasizing setting limits and taking breaks.	☐	5 min	
3	Explain the importance of responsible gambling for financial stability and mental health.	☐	5 min	
4	Highlight the risks of irresponsible gambling, including financial problems, health issues, and social consequences.	☐	5 min	
5	Explain the benefits of responsible gambling, such as reduced stress, improved financial management, and better relationships.	☐	5 min	
6	Summarize key points and provide an activity sheet for participants to reflect on their gambling habits and identify strategies for improvement.	☐	5 min	
7	Discuss the disadvantages of responsible gambling.	☐	5 min	
8	Discuss sources of behavioral confidence in responsible gambling.	☐	5 min	
9	Demonstrate strategies to boost confidence in initiating responsible gambling practices.	☐	5 min	
10	Discuss confidence-boosting strategies for responsible gambling.	☐	5 min	
11	Discuss changes in physical environments that will promote responsible gambling.	☐	5 min	
12	Discuss various responsible gambling tools and resources including their availability and usage.	☐	5 min	
13	Address participants' questions and outline next steps.	☐	3 min	

RESPONSIBLEPLAY©: MTM-based Intervention: Overcoming Barriers to Responsible Gambling - Session Two

Emotional Transformation, Practice for Change, and Changes in the Social Environment

Objective: To evaluate the degree of implementation of the second session of the MTM-based intervention. The program evaluator will utilize this tally sheet to record observed actual behaviors as guided by the planned program.

Observer Name: ______________________

#	Item	Check If Performed	Time Planned	Time upon completion
1	Review key points from the first session, emphasizing the importance of responsible gambling, its benefits, and common barriers.	☐	5 min	
2	Explore feelings related to gambling, both positive and negative, and discuss how these emotions impact gambling behavior.	☐	5 min	
3	Engage participants in brainstorming strategies to overcome barriers to responsible gambling.	☐	5 min	
4	Introduce journal writing as a tool to track gambling activities and emotions.	☐	5 min	
5	Discuss overcoming boredom or frustration associated with responsible gambling practices.	☐	5 min	
6	Conduct an interactive discussion on real-life scenarios and strategies to maintain responsible gambling.	☐	5 min	
7	Present available resources such as counseling services, support groups, and responsible gambling apps.	☐	5 min	
8	Guide participants in identifying a supportive person who can help them maintain responsible gambling behaviors.	☐	5 min	
9	Transform negative emotions associated with gambling into positive emotions through responsible practices.	☐	5 min	
10	Identify new ways to engage in responsible gambling.	☐	5 min	
11	Summarize session content and address participants' questions.	☐	5 min	
12	Outline next steps.	☐	5 min	

RESPONSIBLEPLAY©: MTM-based Intervention: Sustaining Responsible Gambling Behaviors - Session Three

Changes in the Social Environment, Initiation of Responsible Gambling, and Sustenance of Responsible Gambling

Objective: To evaluate the degree of implementation of the third session of the MTM-based intervention. The program evaluator will utilize this tally sheet to record observed actual behaviors as guided by the planned program.

Observer Name: ______________________

#	Item	Check If Performed	Time Planned	Time upon completion
1	Review key points from the second session, emphasizing strategies for overcoming barriers and the importance of support systems.	☐	5 min	
2	Discuss the importance of social support in sustaining responsible gambling behaviors.	☐	10 min	
3	Guide participants in identifying a support person who can help them maintain responsible gambling behaviors.	☐	10 min	
4	Provide participants with various strategies and resources for initiating and maintaining responsible gambling practices.	☐	5 min	
5	Engage participants in brainstorming long-term goals for their responsible gambling behavior.	☐	5 min	
6	Conduct a commitment exercise where participants commit to specific actions for responsible gambling.	☐	5 min	
	Relate to the experiences and anecdotes of a guest speaker from the Nevada Council on Problem Gambling.	☐	5 min	
7	Recap session content, address participants' questions, and outline next steps, including follow-up sessions.	☐	5 min	
8	Provide final encouragement and motivational tips.	☐	5 min	
9	Conclude the session.	☐	5 min	

Knowledge-based Intervention: Introduction to Responsible Gambling - Session One

Title: Understanding Responsible Gambling

Objective: To evaluate the degree of implementation of the first session of the knowledge-based intervention. The program evaluator will utilize this tally sheet to record observed actual behaviors as guided by the planned program.

Observer Name: ______________________

#	Item	Check If Performed	Time Planned	Time upon completion
1	Define responsible gambling, emphasizing setting limits and taking breaks.	☐		
2	Explain the importance of responsible gambling for financial stability and mental health.	☐		
3	List reasons to engage in responsible gambling, including benefits such as reduced stress and better relationships.	☐		
4	Highlight the risks of irresponsible gambling, including financial problems, health issues, and social consequences.	☐		
5	Name and discuss common barriers to practicing responsible gambling, like peer pressure, financial stress, and lack of awareness.	☐		
6	Discuss modifiable risk factors associated with irresponsible gambling behaviors.	☐		
7	Summarize key points and provide an activity sheet for participants to reflect on their gambling habits and identify strategies for improvement.	☐		
8	Address participants' questions and outline next steps.	☐		

Knowledge-based Intervention: Overcoming Barriers to Responsible Gambling - Session Two

Title: Strategies for Responsible Gambling

Objective: To evaluate the degree of implementation of the second session of the knowledge-based intervention. The program evaluator will utilize this tally sheet to record observed actual behaviors as guided by the planned program.

Observer Name: _____________________

#	Item	Check If Performed	Time Planned	Time upon completion
1	Review key points from the first session, emphasizing the importance of responsible gambling, its benefits, and common barriers.	☐		
2	Discuss the psychological and social impacts of gambling.	☐		
3	Discuss the importance of self-control and regulation in gambling.	☐		
4	Discuss the importance of setting time and money limits.	☐		
5	Identify high-risk populations for gambling problems, such as students, low-income individuals, and certain ethnic groups.	☐		
6	List theories used to promote responsible gambling.	☐		
7	Discuss healthy gambling patterns and habits.	☐		
8	Summarize session content and address participants' questions.	☐		
9	Outline next steps.	☐		

Knowledge-based Intervention: Sustaining Responsible Gambling Behaviors - Session Three

Title: Maintaining Responsible Gambling

Objective: To evaluate the degree of implementation of the third session of the knowledge-based intervention. The program evaluator will utilize this tally sheet to record observed actual behaviors as guided by the planned program.

Observer Name: ______________________

#	Item	Check If Performed	Time Planned	Time upon completion
1	Review key points from the second session, emphasizing strategies for overcoming barriers and the importance of support systems.	☐		
2	Discuss the role of family and social support in promoting responsible gambling.	☐		
3	Discuss long-term strategies for maintaining responsible gambling habits, including setting long-term goals and regular self-assessment.	☐		
4	Emphasize the benefits of responsible gambling.	☐		
5	Guide participants in identifying a support person who can help them maintain responsible gambling behaviors.	☐		
6	Provide participants with various strategies and resources for initiating and maintaining responsible gambling practices.	☐		
7	Engage participants in brainstorming long-term goals for their responsible gambling behavior.	☐		
8	Conduct a commitment exercise where participants commit to specific actions for responsible gambling.	☐		
9	Discuss various health promotion theories that support responsible gambling.	☐		
10	Recap session content, address participants' questions, and outline next steps, including follow-up sessions.	☐		
11	Provide final encouragement and motivational tips.	☐		
12	Conclude the session.	☐		

Satisfaction Evaluation Tool

Responsible Gambling Behavior Promotion Program Evaluation

Directions:

Thank you for participating in our Responsible Gambling Behavior Promotion Program. Please help us improve our program by answering the questions below and circling the appropriate response. Your feedback is invaluable.

....

1. Satisfaction with the Program.
    a. Please rate your satisfaction with the following aspects of the program:

Criteria	Excellent	Very Good	Good	Fair	Poor
	(5)	(4)	(3)	(2)	(1)
Information gained from the sessions.	☐	☐	☐	☐	☐
Relevance of the content.	☐	☐	☐	☐	☐
Engagement in session activities.	☐	☐	☐	☐	☐
Effectiveness of the content.	☐	☐	☐	☐	☐
Pace of the sessions.	☐	☐	☐	☐	☐
Methods used by the facilitator.	☐	☐	☐	☐	☐
Time allocated for each session.	☐	☐	☐	☐	☐

....

2. Program Strengths.
    a. Identify two strengths of the program that you would like to highlight:
        i. ____
        ii. ____

....

3. Areas for Improvement.
    a. If you could change something about this program, what would it be?
        i. ____
        ii. ____

....

Thank you for your time!

Management Evaluation Tool

Responsible Gambling Behavior Promotion Program Evaluation

Directions:

Thank you for participating in our Responsible Gambling Behavior Promotion Program. Please help us improve our program by answering the questions below and circling the appropriate response. Your feedback is invaluable.

....

1. How would you rate the administrative support provided?
   ☐ Poor ☐ Fair ☐ Good ☐ Excellent

....

2. How would you rate the communication between program staff and participants?
   ☐ Poor ☐ Fair ☐ Good ☐ Excellent

....

3. How would you rate the adequacy of resources (e.g., time, budget, materials)?
   ☐ Poor ☐ Fair ☐ Good ☐ Excellent

....

4. How effectively were any issues or concerns addressed by the program staff?
   ☐ Poor ☐ Fair ☐ Good ☐ Excellent

....

5. How well does the program adhere to industry best practices for responsible gambling?
   ☐ Poor ☐ Fair ☐ Good ☐ Excellent

....

6. Overall, how satisfied are you with the management of the program?
   ☐ Poor ☐ Fair ☐ Good ☐ Excellent

....

7. How can we improve the management of the program? ______________________

....

8. What do you perceive as the strongest aspect of program management?
   ____

....

Thank you for your time!

Supplement S4: Face And Content Validated Instrument

Measuring Antecedents of Change in Irresponsible Gambling Behavior to Responsible Gambling in University Students

Directions: This survey is voluntary, which means you may choose not to complete it or not to answer individual questions. There is no direct benefit of this survey to you, but your responses will help in developing effective responsible gambling programs. All data from this survey will be kept confidential. **Please put an X mark by the response or fill in the response that correctly describes your position**. Thank you for your help!

…………………………………………………………………………………………………

1. During the past 30 days how many days did you participate in any form of gambling? _______ (Please fill in a value between 0-30)

…………………………………………………………………………………………………

2. Which type of gambling have you participated in the past (choose the one you played the most):

   ☐ Lotteries
   ☐ Instant lotteries
   ☐ Number games (such as Lotto and Keno)
   ☐ Sports betting
   ☐ Horse betting
   ☐ Poker and other card games
   ☐ Casino table games (such as roulette and craps)
   ☐ Bingo
   ☐ Electronic gaming machines (EGMs)
   ☐ Gacha
   ☐ Stock market gambling
   ☐ Playing a skill game for money (e.g. pool or bowling)
   ☐ Fantasy sports
   ☐ Online gambling
   ☐ Video-game based gambling (microtransactions)
   ☐ Other,
   Please specify: ____________________________

…………………………………………………………………………………………………

	Never	Sometimes	Most of the time	Almost always
3. When you think of the past 12 months, have you bet more than you could really afford to lose?	☐	☐	☐	☐
4. Still thinking about the last 12 months, have you needed to gamble with larger amounts of money to get the same feeling of excitement?	☐	☐	☐	☐
5. When you gambled, did you go back another day to try to win back the money you lost?	☐	☐	☐	☐
6. Have you borrowed money or sold anything to get money to gamble?	☐	☐	☐	☐
7. Have you felt that you might have a problem with gambling?	☐	☐	☐	☐
8. Has gambling caused you any health problems, including stress or anxiety?	☐	☐	☐	☐
9. Have people criticized your betting or told you that you had a gambling problem, regardless of whether or not you thought it was true?	☐	☐	☐	☐
10. Has your gambling caused any financial problems for you or your household?	☐	☐	☐	☐
11. Have you felt guilty about the way you gamble or what happens when you gamble?	☐	☐	☐	☐

…………………………………………………………………………………………………

For this study, we define <u>responsible gambling</u> as:
- Taking regular breaks from gambling.
- Not relying on gambling as a source of income.
- Only gambling with money, you can afford to lose.
- Setting personal limits on both time and money spent on gambling.

Instructions for questions #12 to #43:
<u>Please choose the best option that reflects how much you believe each outcome will occur following responsible gambling.</u>
…………………………………………………………………………………………………

	Not at all likely	Somewhat likely	Moderately likely	Very likely	Completely likely
12. If you participate in responsible gambling, you will be financially stable.	☐	☐	☐	☐	☐
13. If you participate in responsible gambling, you will perform well in your studies.	☐	☐	☐	☐	☐
14. If you participate in responsible gambling, you will have good mental health.	☐	☐	☐	☐	☐

15. If you participate in responsible gambling, you will have good social relationships.	☐	☐	☐	☐	☐
16. If you participate in responsible gambling, you will develop positive skills (such as greater observation skills, challenging your mind, and analyzing patterns and numbers) from gambling.	☐	☐	☐	☐	☐

....

	Not at all likely	Somewhat likely	Moderately likely	Very likely	Completely likely
17. If you participate in responsible gambling, you will find it takes too much effort to keep up with responsible gambling skills.	☐	☐	☐	☐	☐
18. If you participate in responsible gambling, you will have urges to gamble.	☐	☐	☐	☐	☐
19. If you participate in responsible gambling, you will feel excluded from gambling-related social activities.	☐	☐	☐	☐	☐
20. If you participate in responsible gambling, you will miss the rush of excitement that comes with gambling.	☐	☐	☐	☐	☐
21. If you participate in responsible gambling, you will feel pressured or anxious about maintaining responsible gambling habits.	☐	☐	☐	☐	☐

....

	Not at all confident	Slightly confident	Moderately confident	Very confident	Completely confident
22. How confident are you that you can participate in responsible gambling, most of the time in the next week?	☐	☐	☐	☐	☐
23. How confident are you that you can participate in responsible gambling, when others are gambling irresponsibly around you in the next week?	☐	☐	☐	☐	☐

24. How confident are you that you can participate in responsible gambling, when you have urges to gamble in the next week?	☐	☐	☐	☐	☐
25. How confident are you that you can participate in responsible gambling, when others ask you to gamble with them in the next week?	☐	☐	☐	☐	☐

…………………………………………………………………………………………………

For the next three question (26-28) responsible gambling strategies example are as follows:

- Taking regular breaks from gambling.
- Not relying on gambling as a source of income.
- Only gambling with money, you can afford to lose.
- Setting personal limits on both time and money spent on gambling.

…………………………………………………………………………………………………

	**Not at all sure**	**Slightly sure**	**Moderately sure**	**Very sure**	**Completely sure**
26. How sure are you that you will not visit a place where gambling is going on, after exhausting all your responsible gambling strategies?	☐	☐	☐	☐	☐
27. How sure are you that you will not be around people who gamble, after exhausting all your responsible gambling strategies?	☐	☐	☐	☐	☐
28. How sure are you that you will be able to stop accessing your money for gambling, after exhausting all your responsible gambling strategies?	☐	☐	☐	☐	☐

…………………………………………………………………………………………………

	**Not at all sure**	**Slightly sure**	**Moderately sure**	**Very sure**	**Completely sure**
29. How sure are you that you can control the urge to gamble?	☐	☐	☐	☐	☐
30. How sure are you that you can motivate yourself to participate in responsible gambling?	☐	☐	☐	☐	☐
31. If you were to experience doubt about your ability to engage in responsible gambling, how sure are you that you could overcome this feeling?	☐	☐	☐	☐	☐

…………………………………………………………………………………………………

	Not at all sure	Slightly sure	Moderately sure	Very sure	Completely sure
32. How sure are you that you can keep a self-record to monitor your gambling behavior?	☐	☐	☐	☐	☐
33. How sure are you that you can participate in responsible gambling behavior if you encounter barriers such as peer pressure?	☐	☐	☐	☐	☐
34. How sure are you that you can change your plan to participate in responsible gambling if you face financial difficulties?	☐	☐	☐	☐	☐

………………………………………………………………………………………………

	Not at all sure	Slightly sure	Moderately sure	Very sure	Completely sure
35. How sure are you that you can get the help of a family member to support you in participating in responsible gambling?	☐	☐	☐	☐	☐
36. How sure are you that you can get the help of a friend to support you in participating in responsible gambling?	☐	☐	☐	☐	☐
37. How sure are you that you can get the help of a health professional to support you in participating in responsible gambling?	☐	☐	☐	☐	☐

………………………………………………………………………………………………

	Not at all likely	Somewhat likely	Moderately likely	Very likely	Completely likely
38. How likely is it that you will participate in responsible gambling in the upcoming week?	☐	☐	☐	☐	☐
39. How likely is it that you want to participate in responsible gambling in the upcoming week?	☐	☐	☐	☐	☐
40. How likely is it that you intend to participate in responsible gambling in the upcoming week?	☐	☐	☐	☐	☐

………………………………………………………………………………………………

	Not at all likely	Somewhat likely	Moderately likely	Very likely	Completely likely

41. How likely is it that you will participate in responsible gambling every day from now on?	☐	☐	☐	☐	☐
42. How likely is it that you want to participate in responsible gambling every day from now on?	☐	☐	☐	☐	☐
43. How likely is it that you intend to participate in responsible gambling every day from now on?	☐	☐	☐	☐	☐

....

44. Do you think you have a gambling problem?

☐ Yes
☐ No

....

45. How often do you feel that your substance use (including alcohol, recreational drugs, and tobacco) has affected your gambling behavior?

☐ Never
☐ Rarely
☐ Sometimes
☐ Often
☐ Always
☐ Not applicable

....

46. How often do you find that your mental health affects your gambling decisions?

☐ Never
☐ Rarely
☐ Sometimes
☐ Often
☐ Always
☐ Not applicable

....

47. What sex were you assigned at birth (on your original birth certificate)?

☐Male
☐Female

48. How old are you today? ___________________________ years

49. What is your race or ethnicity?

☐ American Indian / Alaska Native, Alaskan
☐ Asian, Asian American
☐ Biracial, multiracial, of mixed race
☐ Black and African American
☐ Hispanic, Latino/a, Latinx, Latine
☐ Middle East, Middle Eastern or North African, Arab Americans
☐ White
☐Other,
Please specify: ____________________________

50. What is your class?

☐ Freshmen
☐ Sophomore
☐ Junior
☐ Senior
☐ Masters
☐ Doctoral
☐ Professional

51. What is your current overall GPA? (on a 4.00 scale)

☐ Less than 1.99
☐ 2.00 – 2.49
☐ 2.50 – 2.99
☐ 3.00 – 3.49
☐ 3.50 – 4.00

52. Where do you live?

☐ On campus
☐ Off-campus

....

53. Do you work?

☐ No
☐ Yes,
_______________ average hours per well (put a number, not a range)

....

54. Is your work related to the hotel or hospitality industry?

☐ No
☐ Yes

....

Thank you for your time!

SCORING (PGSI)

Scale: Never (0), Sometimes (1), Most of the time (2), Almost always (3). A summative score of Items 3-11. Possible range: 0- 27. The higher your score, the greater the risk that your gambling is a problem.

A score of 0: Non-problem gambling.
A Score of 1 or 2: Low level of problems with few or no identified negative consequences.
A score of 3 to 7: Moderate level of problems leading to some negative consequences.
A score of 8 or more: Problem gambling with negative consequences and a possible loss of control.

Flesch Reading Ease: 73.8
Flesch-Kincaid Grade Level: 6.9

Ferris, J., & Wynne, H. (2001). The Canadian problem gambling index: Final report. Submitted for the Canadian Centre on Substance Abuse.

SCORING (MTM)

The construct of advantages: Scale: Not at all likely (0), Somewhat likely (1), Moderately likely (2), Very likely (3), Completely likely (4). A summative score of Items 12-16. Possible range: 0-20. The high score is associated with the likelihood of initiation of behavior change.

The construct of disadvantages: Scale: Not at all likely (0), Somewhat likely (1), Moderately likely (2), Very likely (3), Completely likely (4). A summative score of Items 17-21. Possible range: 0-20. The low score is associated with the likelihood of initiation of behavior change. Subtract the disadvantages score from the advantages scores to calculate the **participatory dialogue** construct score. Possible range: -20 - +20. A positive score will be indicative of behavior change.

The construct of behavioral confidence: Scale: Not at all confident (0), slightly confident (1), moderately confident (2), very confident (3), completely confident (4). A summative score of Items 22-25. Possible range 0-16. The high score is associated with the likelihood of initiation of behavior change.

The construct of changes in the physical environment: Scale: Not at all sure (0), slightly sure (1), moderately sure (2), very sure (3), completely sure (4). A summative score of Items 26-28. Possible range 0-12. The high score is associated with the likelihood of initiation of behavior change.

The construct of emotional transformation: Scale: Not at all sure (0), slightly sure (1), moderately sure (2), very sure (3), completely sure (4). A summative score of Items 29-31. Possible range 0-12. The high score is associated with the likelihood of sustenance of behavior change.

The construct of practice for change: Scale: Not at all sure (0), slightly sure (1), moderately sure (2), very sure (3), completely sure (4). The summative score of Items 32-34. Possible range 0-12. The high score is associated with the likelihood of sustenance of behavior change.

The construct of changes in the social environment: Scale: Not at all sure (0), slightly sure (1), moderately sure (2), very sure (3), completely sure (4). A summative score of Items 35-37. Possible range 0-12. The high score is associated with the likelihood of sustenance of behavior change.

The construct of intention to initiate responsible gambling: Scale: not at all likely (0), somewhat likely (1), moderately likely (2), very likely (3), and completely likely (4). The summative score of Items 38-40. Possible range: 0-12. This can be the dependent variable in multiple linear regression modeling for the initiation model.

The construct of intention to sustain responsible gambling: Scale: not at all likely (0), somewhat likely (1), moderately likely (2), very likely (3), and completely likely (4). The summative score of Items 41-43. Possible range: 0-12. This can be the dependent variable in multiple linear regression modeling for the sustenance model.

Flesch Reading Ease: 52.7 | Flesch-Kincaid Grade Level: 9.8
